# Supplementary material for: Co-transcriptional splicing efficiency is a gene-specific feature that can be regulated by TGFβ
Source: Commun Biol. 2022 Mar 28;5:277. doi: 10.1038/s42003-022-03224-z (PMC8960766; doi:10.1038/s42003-022-03224-z)
Supplement: Supplementary file 2 — Supplementary Information [file 42003_2022_3224_MOESM2_ESM.pdf]

Co-transcriptional splicing efficiency is a gene-specific feature that can be regulated by TGFβ

Elena Sánchez-Escabias, Jose A. Guerrero-Martínez[*] and Jose C. Reyes[*]

Centro Andaluz de Biología Molecular y Medicina Regenerativa-CABIMER. Consejo Superior de Investigaciones Científicas-Universidad de Sevilla-Universidad Pablo de Olavide (CSIC-USE-UPO). Av. Americo Vespucio 41092 Seville, Spain.

Supplementary Figures and Tables.

Supplementary figure 1.
Supplementary figure 2.
Supplementary figure 3.
Supplementary figure 4.
Supplementary figure 5.
Supplementary figure 6
Supplementary figure 7
Supplementary Table 1.

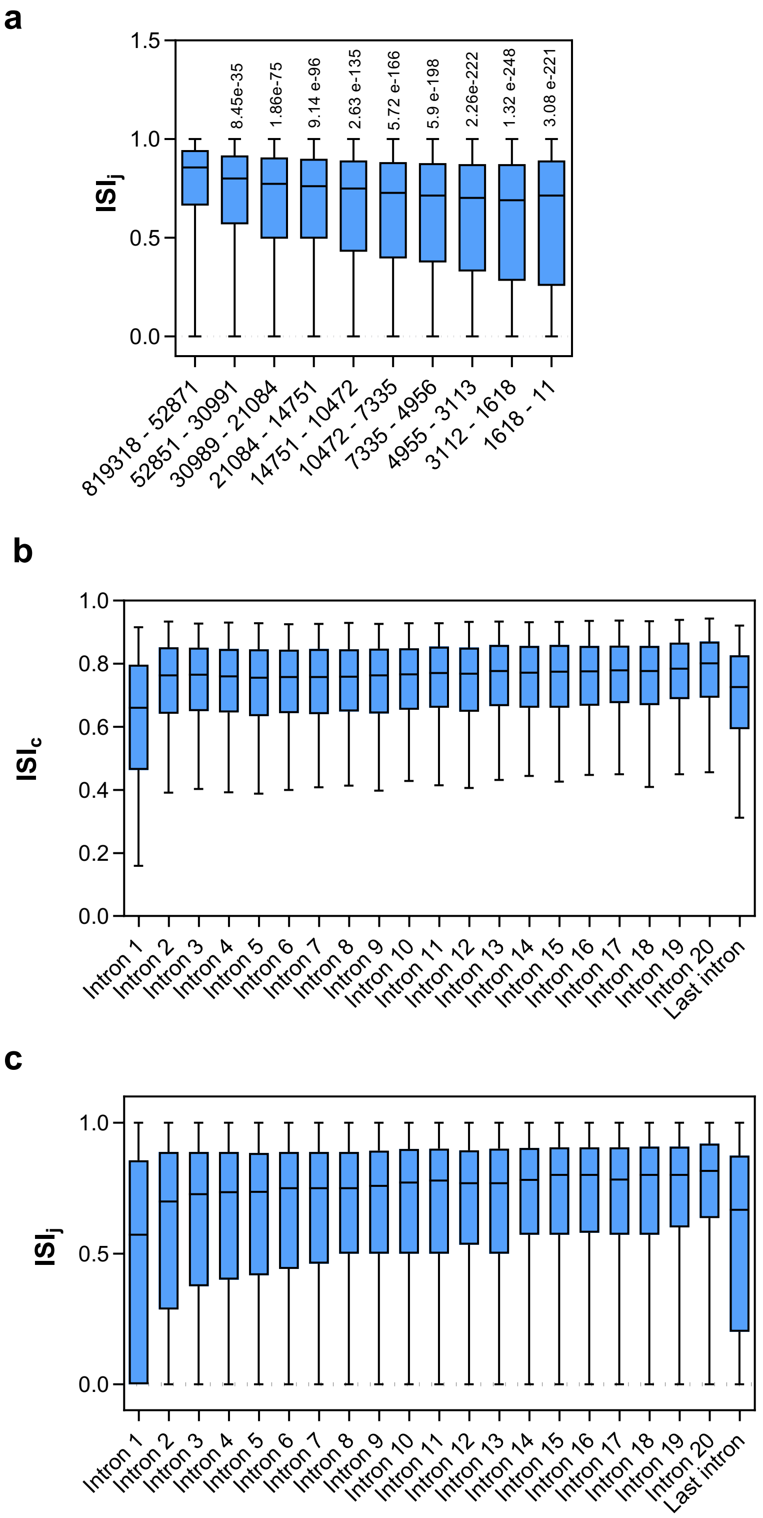

Supplementary Fig. 1. a Effect of intron to polyA site distance on ISI_f. Introns were divided into ten deciles depending on their distance to the polyA site. Unpaired Student's *t*-test p-values of the indicated decile with respect to the first decile are shown. **b** Boxplot of ISI_c levels of the first twenty introns and the last intron of all expressed genes. **c** Boxplot of ISI_j level of the first twenty introns and the last intron of all expressed genes. Sample size of all set of data are provided in Supplementary Data 4.

Supplementary Figure 1

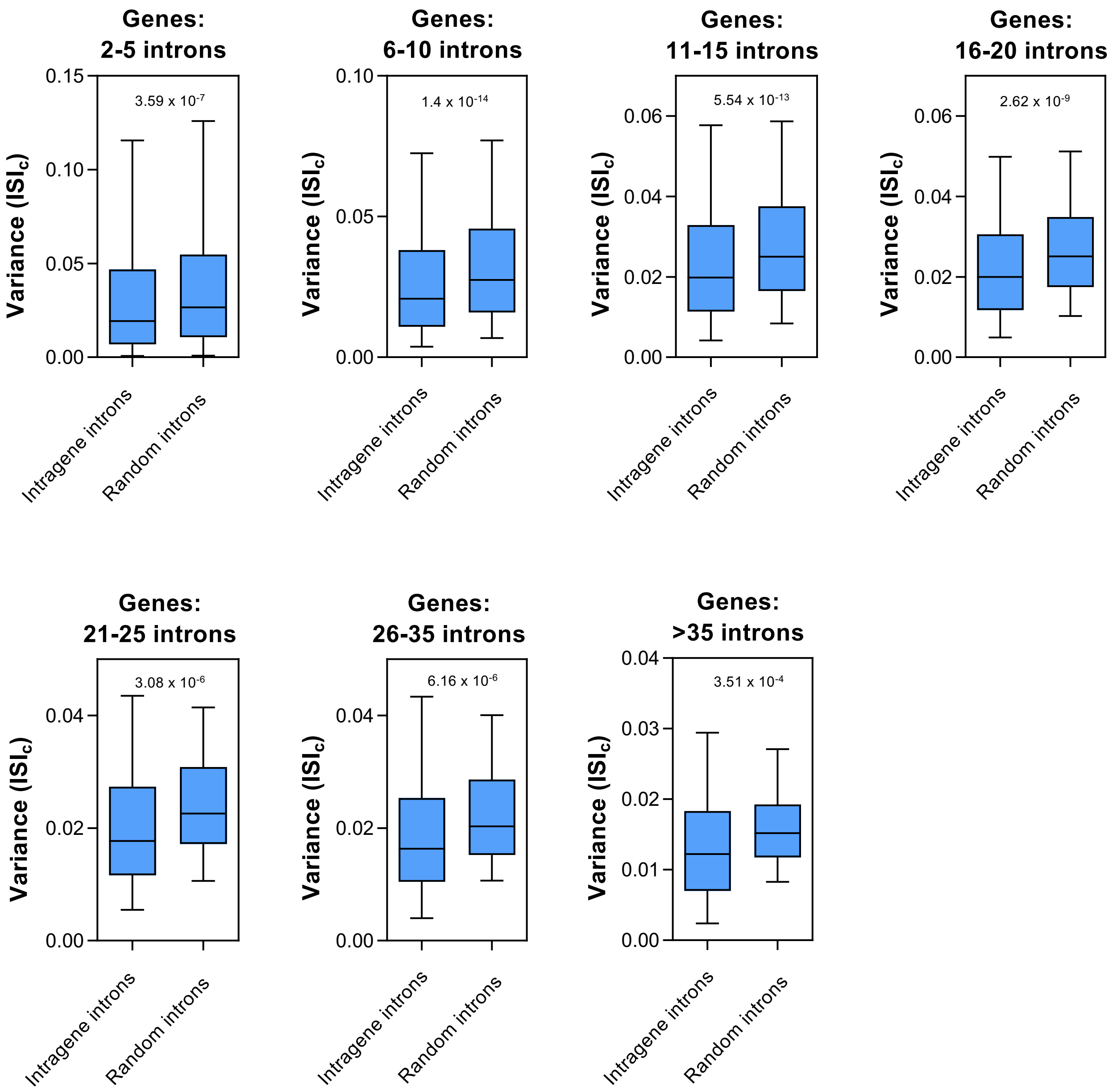

Supplementary Fig. 2. Variance of ISI_c values across introns within the same gene or the same number of randomly sampled introns. In each plot only genes with the indicated number of introns were used. Unpaired Student`s *t*-test p-values are shown. Sample size of all set of data are provided in Supplementary Data 4.

Supplementary Figure 2

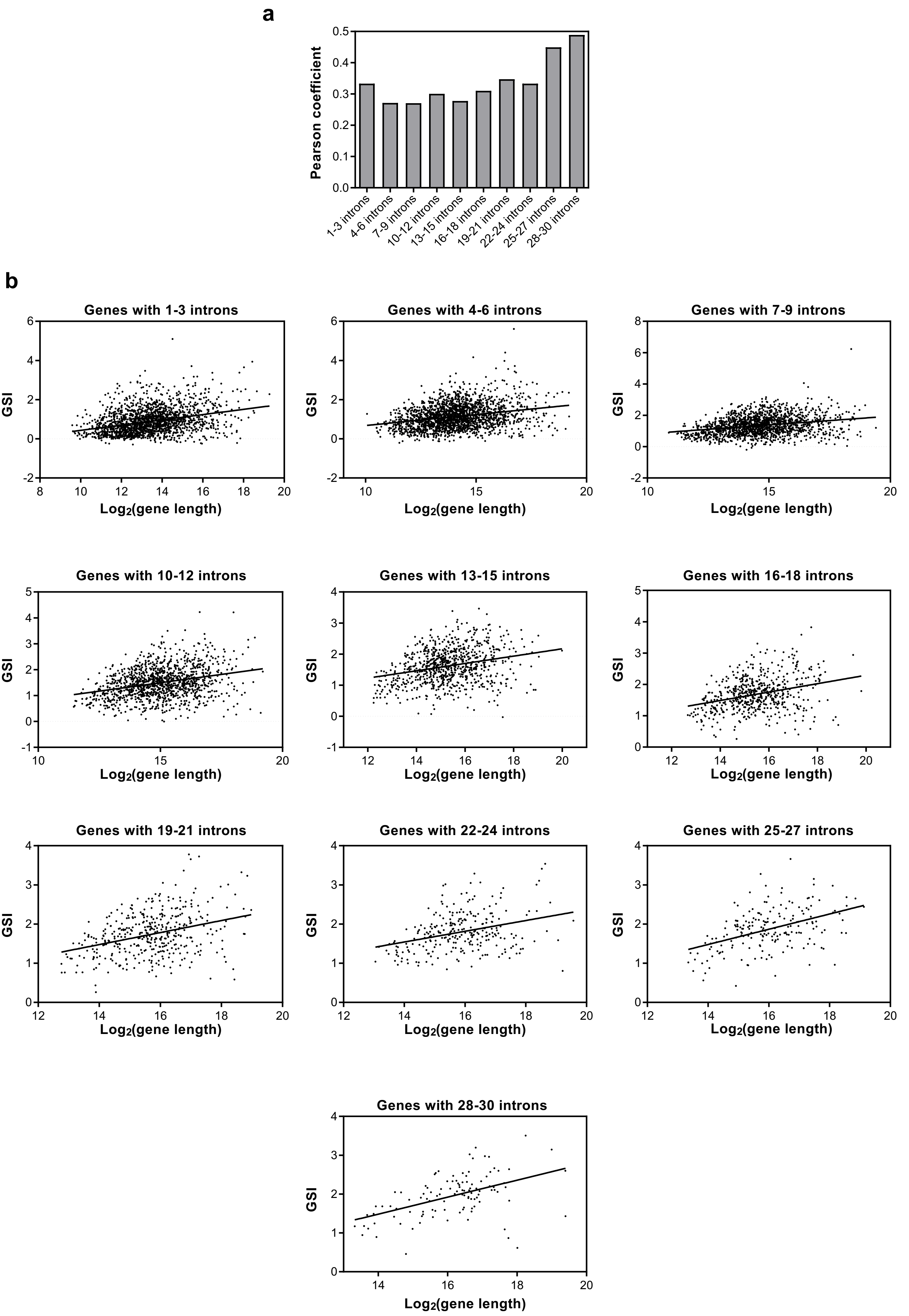

Supplementary Figure 3

Supplementary Fig. 3. a Pearson correlation between gene length and GSI depending on intron number. Expressed genes were clustered into ten groups depending on their intron number (from 1 to 30 introns), and Pearson correlation coefficients within each group were calculated. **b** Correlation plots and regression line between gene length and GSI of genes with different numbers of introns (1–3, 4–6, 7–9, 10–12, 13–15, 16–18, 19–21, 22–24, 25–27 or 28–30). Pearson correlation coefficient of the plots are shown in (**a**). Sample size of all set of data are provided in Supplementary Data 4.

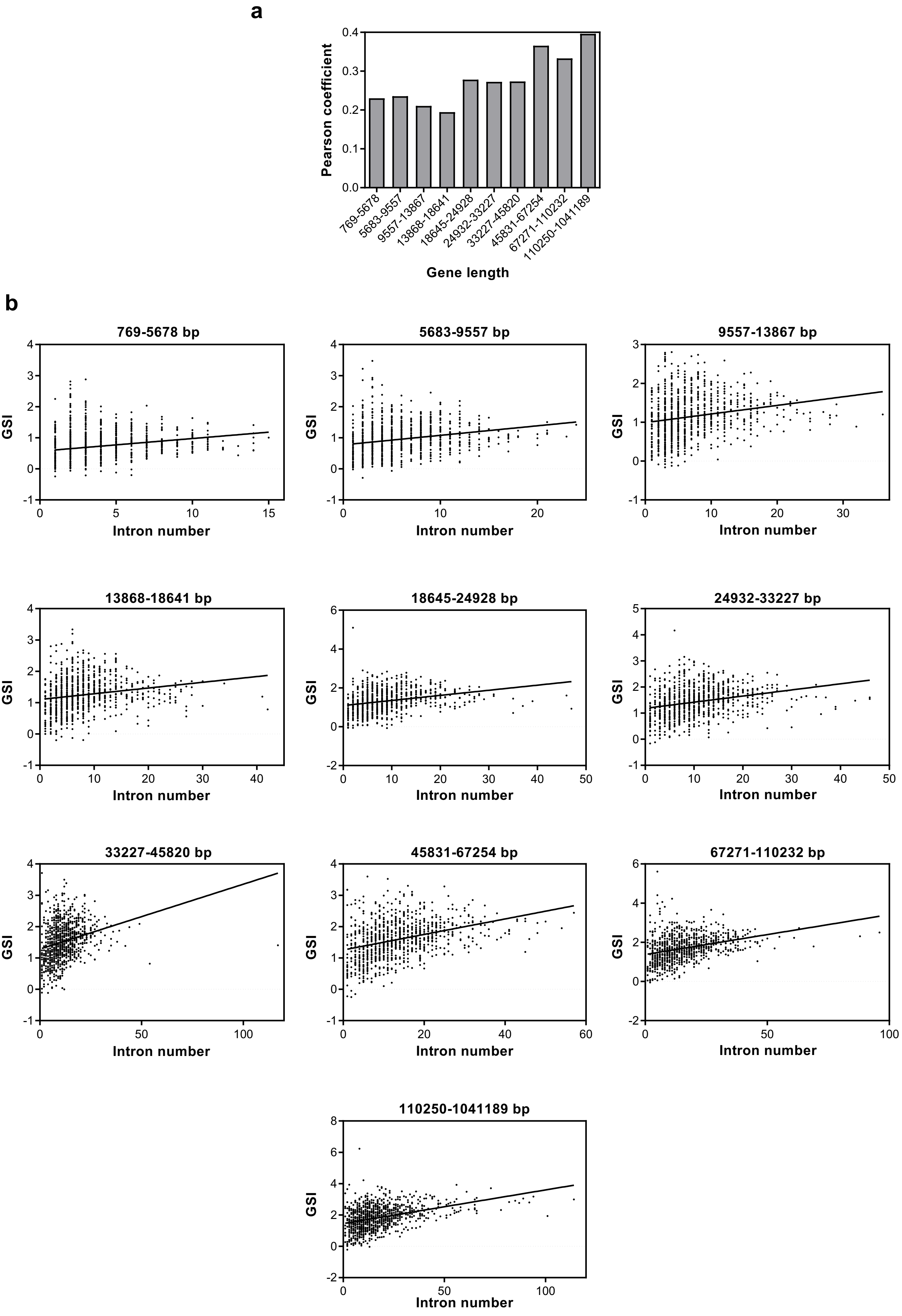

Supplementary Figure 4

Supplementary Fig. 4. a Pearson correlation between intron number and GSI depending on gene length. Expressed genes were clustered into ten groups depending on their length, and Pearson correlation coefficients within each group were calculated. **b** Correlation plots and regression line between intron number and GSI of all genes clustered into ten bins according to their length: 769–5678 bp, 5683–9557 bp, 9557–13867 bp, 13868–18641 bp, 18645–24928 bp, 24932–33227 bp, 33227–45820 bp, 45831–67254 bp, 67271–110232 bp and 110250–1041189 bp. Pearson correlation coefficient of the plots are shown in **(a).** bp, basepairs. Sample size of all set of data are provided in Supplementary Data 4.

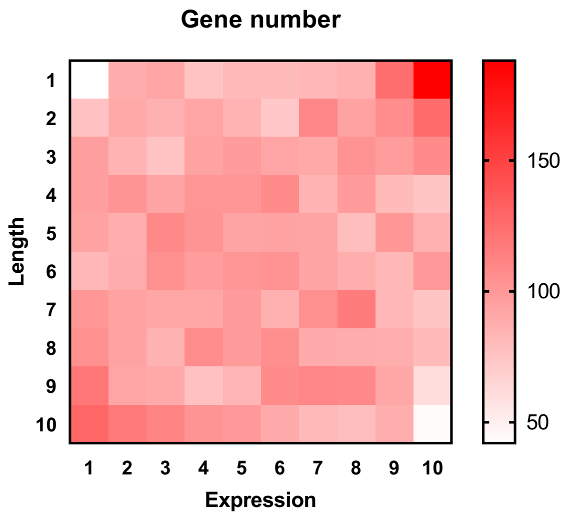

Supplementary Fig. 5. Heatmap showing of number of genes of the matrix L × E used in Fig. 3G–J. To construct the matrix, the expressed genes population was divided into ten bins according to gene length (L_i, with i = 1, 2, …10) and into another ten binds according to pre-mRNA levels (E_j, with j = 1, 2, …10). A matrix (L × E) was then constructed by assigning genes to the corresponding positions $a_{i,j}$ according to their respective length (L_i) and pre-mRNA level (E_j).

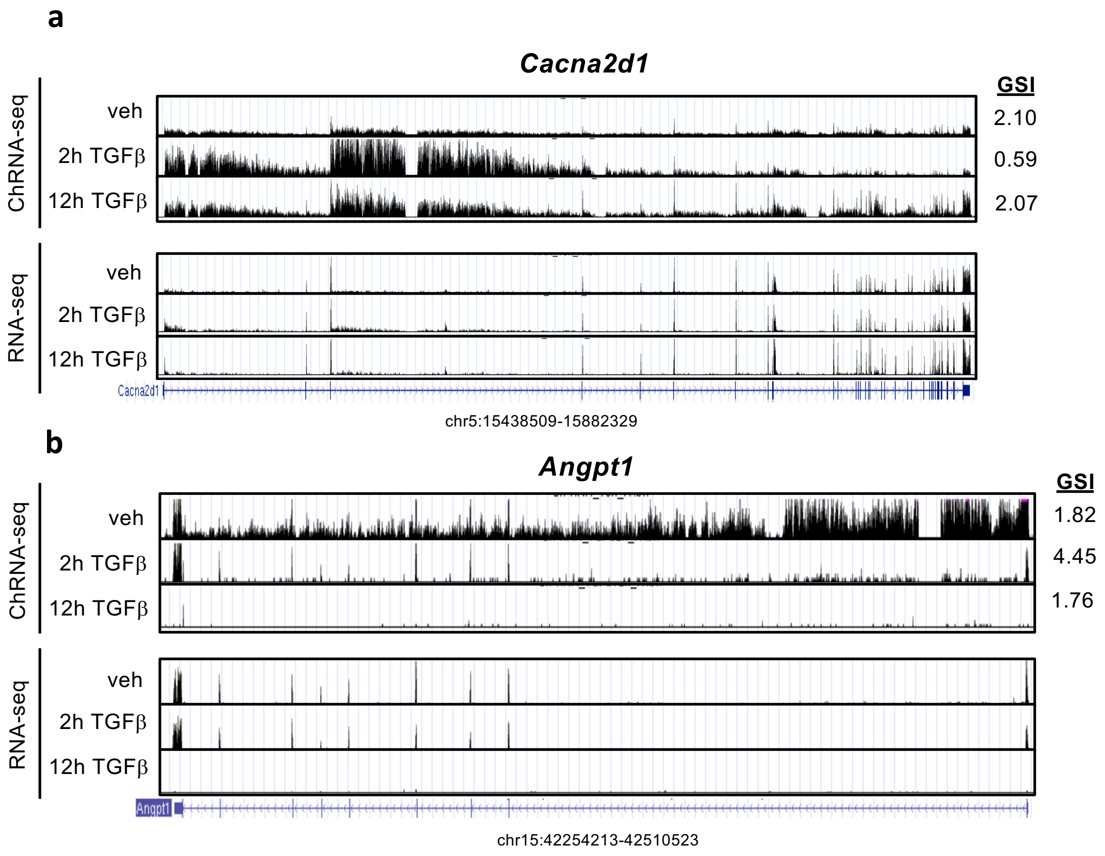

Supplementary Fig. 6. a, b ChrRNA-seq and RNA-seq IGV snapshot of *Cacna2d1* **(a)** and *Angpt1* **(b)** in vehicle or at 2 h or 12 h after TGFβ treatment.

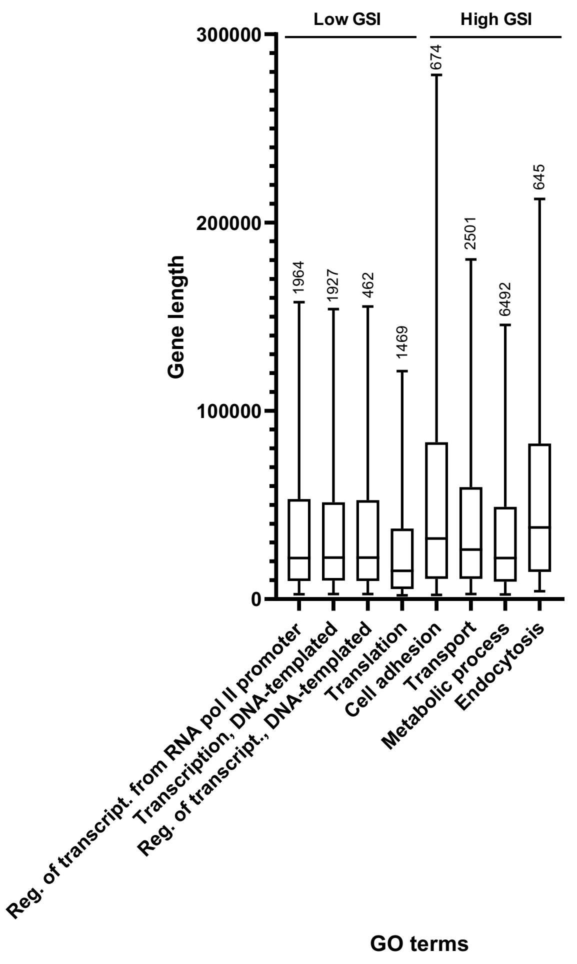

Supplementary Fig. 7. Boxplots showing gene length distributions of genes of the indicated GO terms. Number of genes in each term is indicated.

Supplementary Table1

	Type	Forward	Reverse
Id3_exon	Exon-Exon	ACTCAGCTTAGCCAGGTGGA	GAGATCACAAGTTCCGGAGTG
Id3_intron	Intron-Intron	GAGGAGGGAGGCTTGCAT	TTTCAGTGGCTTGGCTTTTT
Inadl_exon	Exon-Exon	CCCTGGACTCAAAAGTCAGC	CTCAGAGCCACCACACTGAA
Inadl_intron	Intron-Intron	CAGCGTCAGTTCTGTGGAAA	AGGGTGGGAAAACAGAAACC
Utrn_exon	Exon-Exon	ATCTTCTCGAAGGCCTCACA	CTTGTGGAACCACGTTCCTT
Utrn_intron_2	Intron-Intron	CCTGGTTGCCTCCTTTTCTT	AGGGCCTGAAAGTCAACAGA
Utrn_intron_22	Intron-Intron	ATGGGATGGTGTGCCAGTAT	TCAAACTGGCCACTAAAGGTG
Wdr1_exon	Exon-Exon	TGCTCCTTCTGTGTGGTATCC	TGCTCCTTCTGTGTGGTATCC
Wdr1_intron	Exon-Intron	TGCTCCTTCTGTGTGGTATCC	TGCTCCTTCTGTGTGGTATCC
Csrp1_exon	Exon-Exon	CAGACAAGGGGGAGTCTCTG	ATCTTCTGGGCAAACTTGGA
Csrp1_intron	Exon-Intron	CAGACAAGGGGGAGTCTCTG	AAAGGTGTTTCGTCGGTGTC
