# Supplementary material for: Co-transcriptional splicing efficiency is a gene-specific feature that can be regulated by TGFβ
Source: Commun Biol. 2022 Mar 28;5:277. doi: 10.1038/s42003-022-03224-z (PMC8960766; doi:10.1038/s42003-022-03224-z)
Supplement: Supplementary file 3 — Description of Additional Supplementary Files [file 42003_2022_3224_MOESM3_ESM.pdf]

Description of Additional Supplementary Files

File name: Supplementary Data 1.

Description: List of junction-based Intron splicing Index ISIj and coverage-based Intron splicing Index ISIc values for all analyzed introns.

File name: Supplementary Data 2.
Description: List of Gene Splicing Index (GSI) values for all expressed genes.

File name: Supplementary Data 3.

Description: List of differential GSI values (ΔGSI) at 2 h or 12 h versus vehicletreated (control) cells.

File name: Supplementary Data 4.

Description: Sample size (n) used to derive statistics for all set of data.

File name: Supplementary Data 5.

Description: Source data for all graphs and charts.
