# Supplementary material for: Co-transcriptional splicing efficiency is a gene-specific feature that can be regulated by TGFβ
Source: Commun Biol. 2022 Mar 28;5:277. doi: 10.1038/s42003-022-03224-z (PMC8960766; doi:10.1038/s42003-022-03224-z)
Supplement: Supplementary file 9 — Reporting Summary [file 42003_2022_3224_MOESM9_ESM.pdf]

nature portfolio

Corresponding author(s): Jose C. Reyes
Jose A. Guerrero-Martinez
COMMSBIO-21-2009

Last updated by author(s): Feb 17, 2022

Reporting Summary

Nature Portfolio wishes to improve the reproducibility of the work that we publish. This form provides structure for consistency and transparency in reporting. For further information on Nature Portfolio policies, see our Editorial Policies and the Editorial Policy Checklist.

Statistics

For all statistical analyses, confirm that the following items are present in the figure legend, table legend, main text, or Methods section.

n/a	Confirmed	
☐	☒	The exact sample size (*n*) for each experimental group/condition, given as a discrete number and unit of measurement
☐	☒	A statement on whether measurements were taken from distinct samples or whether the same sample was measured repeatedly
☐	☒	The statistical test(s) used AND whether they are one- or two-sided<br>*Only common tests should be described solely by name; describe more complex techniques in the Methods section.*
☒	☐	A description of all covariates tested
☐	☒	A description of any assumptions or corrections, such as tests of normality and adjustment for multiple comparisons
☐	☒	A full description of the statistical parameters including central tendency (e.g. means) or other basic estimates (e.g. regression coefficient) AND variation (e.g. standard deviation) or associated estimates of uncertainty (e.g. confidence intervals)
☒	☐	For null hypothesis testing, the test statistic (e.g. *F*, *t*, *r*) with confidence intervals, effect sizes, degrees of freedom and *P* value noted<br>*Give P values as exact values whenever suitable.*
☒	☐	For Bayesian analysis, information on the choice of priors and Markov chain Monte Carlo settings
☒	☐	For hierarchical and complex designs, identification of the appropriate level for tests and full reporting of outcomes
☐	☒	Estimates of effect sizes (e.g. Cohen's *d*, Pearson's *r*), indicating how they were calculated

Our web collection on statistics for biologists contains articles on many of the points above.

Software and code

Policy information about availability of computer code

Data collection	No software was used for data collection
Data analysis	Software version source<br>R v3.4.4 https://cran.r-project.org/bin/linux/ubuntu/<br>RStudio v0.99.879 https://rstudio.com/products/rstudio/download/<br>RSubread v1.28.1 https://bioconductor.org/packages/release/bioc/html/Rsubread.html<br>Bioconductor v2.38 https://www.bioconductor.org/install/<br>Stringtie. https://ccb.jhu.edu/software/stringtie/<br>GffCompare. https://ccb.jhu.edu/software/stringtie/gffcompare.shtml<br>squid.py (https://github.com/Xinglab/SQUID)<br>DAVID tools (https://david.ncifcrf.gov/).<br>Limma-Voom (v.3.34.9). https://ucdavis-bioinformatics-training.github.io/2018-June-RNA-Seq-Workshop/thursday/DE.html<br>edgeR (v.3.20.9). https://bioconductor.org/packages/release/bioc/html/edgeR.html<br>https://www.rdocumentation.org/packages/edgeR/versions/3.14.0/topics/calcNormFactors<br>rasterVis. https://cran.r-project.org/web/packages/rasterVis/rasterVis.pdf<br>RnetCDF(). https://www.unidata.ucar.edu/software/netcdf/<br>bedtools v2.27.1 https://bedtools.readthedocs.io/en/latest/content/installation.html

For manuscripts utilizing custom algorithms or software that are central to the research but not yet described in published literature, software must be made available to editors and reviewers. We strongly encourage code deposition in a community repository (e.g. GitHub). See the Nature Portfolio guidelines for submitting code & software for further information.

Data

Policy information about availability of data

All manuscripts must include a data availability statement. This statement should provide the following information, where applicable:

- Accession codes, unique identifiers, or web links for publicly available datasets
- A description of any restrictions on data availability
- For clinical datasets or third party data, please ensure that the statement adheres to our policy

The datasets supporting the conclusions of this article are included within the article (Guerrero-Martinez et al., 2020), and the data are available in GEO accession number GSE140552

Field-specific reporting

Please select the one below that is the best fit for your research. If you are not sure, read the appropriate sections before making your selection.

☒ Life sciences ☐ Behavioural & social sciences ☐ Ecological, evolutionary & environmental sciences

For a reference copy of the document with all sections, see nature.com/documents/nr-reporting-summary-flat.pdf

Life sciences study design

All studies must disclose on these points even when the disclosure is negative.

Sample size	Samples size for each experiment is indicated in the figure legends. The sample size was chosen based on previous experience in the lab, for each experiment to yield high statistics power. For RT-qPCR n = 4 was used.<br>Our sample sizes are similar to those generally employed in other relevant similar studies:<br>Guo, H., Ci, X., Ahmed, M. et al, 2019. https://doi.org/10.1038/s41467-018-08133-6<br>Fox, S., Myers, J.A. et al., 2020. https://pubmed.ncbi.nlm.nih.gov/32917861/<br>Li, J., Huang, K., Hu, G. et al, 2019. https://doi.org/10.1038/s41467-019-08949-w<br>No statistical methods were used to predetermine sample size.
Data exclusions	No data were excluded from the analyses
Replication	RT-qPCR experiments were performed in at least 4 biologically independent replicates using two or three technical replicates. Technical replicates were used only to determine value of each independent biological replicate, but not to calculate the final statistics, and were not quantified to stablish the n value. All attempts at replication of the results were successful.
Randomization	This study does not involve randomization of samples, following standard procedures generally employed in other relevant similar studies. See for example Partridge et al., 2020 (https://www.nature.com/articles/s41586-020-2023-4) or Douillet et al., 2020 (https://www.nature.com/articles/s41588-020-0618-1)
Blinding	Investigators were not blinded for selection of cell culture plates used in the treatments (vehicle-treated or TGFbeta-treated) following standard procedures generally employed in other relevant similar studies. See for example Partridge et al., 2020 (https://www.nature.com/articles/s41586-020-2023-4) or Douillet et al., 2020 (https://www.nature.com/articles/s41588-020-0618-1).

Reporting for specific materials, systems and methods

We require information from authors about some types of materials, experimental systems and methods used in many studies. Here, indicate whether each material, system or method listed is relevant to your study. If you are not sure if a list item applies to your research, read the appropriate section before selecting a response.

Materials & experimental systems

n/a	Involved in the study	
☒	☐	Antibodies
☐	☒	Eukaryotic cell lines
☒	☐	Palaeontology and archaeology
☒	☐	Animals and other organisms
☒	☐	Human research participants
☒	☐	Clinical data
☒	☐	Dual use research of concern

Methods

n/a	Involved in the study	
☒	☐	ChIP-seq
☒	☐	Flow cytometry
☒	☐	MRI-based neuroimaging

Eukaryotic cell lines

Policy information about cell lines

Cell line source(s)	NMuMG cell line was provided by Jose Antonio Pintor-Toro (commercial source: ATCC)
Authentication	NMuMG was validated as a mouse cell line through our Next Generation Sequencing experiments. Moreover, both the epithelial phenotype and the kinetic of the induction to the mesenchymal phenotype with TGFbeta were assessed in our experiments many times through immunofluorescence, western blotting and RT-qPCR analysis of epithelial to mesenchymal transition markers assays (SNAI1, VIM, FN1, CDH1). All these characteristics are typical and some of them unique of NMuMG cells.
Mycoplasma contamination	All cell lines were tested negative for mycoplasma contamination
Commonly misidentified lines (See ICLAC register)	No commonly misidentified cell lines was used in this study
